# Reactive oxygen species induce expression of vascular endothelial growth factor in chondrocytes and human articular cartilage explants

**DOI:** 10.1186/ar2102

**Published:** 2006-12-23

**Authors:** Jakob Fay, Deike Varoga, Christoph J Wruck, Bodo Kurz, Mary B Goldring, Thomas Pufe

**Affiliations:** 1 Department of Trauma Surgery, University Hospital Schleswig-Holstein, Campus Kiel, Arnold-Heller-Strasse 7, 24105 Kiel, Germany; 2 Department of Anatomy, Christian-Albrechts-University Kiel, Olshausenstrasse 40, 24098 Kiel, Germany; 3 Beth Israel Deaconess Medical Center, New England Baptist Bone and Joint Institute, Harvard Institutes of Medicine, HIM-246, 4 Blackfan Circle, Boston, MA 02115-5713, USA; 4 Hospital for Special Surgery, Caspary Research Building, Room 528, 535 E. 70th Street, New York, NY 10021, USA

## Abstract

Vascular endothelial growth factor (VEGF) promotes cartilage-degrading pathways, and there is evidence for the involvement of reactive oxygen species (ROS) in cartilage degeneration. However, a relationship between ROS and VEGF has not been reported. Here, we investigate whether the expression of VEGF is modulated by ROS.

Aspirates of synovial fluid from patients with osteoarthritis (OA) were examined for intra-articular VEGF using ELISA. Immortalized C28/I2 chondrocytes and human knee cartilage explants were exposed to phorbol myristate acetate (PMA; 0–20 μg/ml), which is a ROS inducer, or 3-morpholino-sydnonimine hydrochloride (SIN-1; 0–20 μM), which is a ROS donor. The levels of VEGF protein and nitric oxide (NO) production were determined in the medium supernatant, using ELISA and Griess reagent, respectively. Gene expression of VEGF-121 and VEGF-165 was determined by splice variant RT-PCR. Expression of VEGF and VEGF receptors (VEGFR-1 and VEGFR-2) was quantified by real-time RT-PCR.

Synovial fluid from OA patients revealed markedly elevated levels of VEGF. Common RT-PCR revealed that the splice variants were present in both immortalized chondrocytes and cartilage discs. In immortalized chondrocytes, stimulation with PMA or SIN-1 caused increases in the levels of VEGF, VEGFR-1 and VEGFR-2 mRNA expression. Cartilage explants produced similar results, but VEGFR-1 was only detectable after stimulation with SIN-1. Stimulation with PMA or SIN-1 resulted in a dose-dependent upregulation of the VEGF protein (as determined using ELISA) and an increase in the level of NO in the medium.

Our findings indicate ROS-mediated induction of VEGF and VEGF receptors in chondrocytes and cartilage explants. These results demonstrate a relationship between ROS and VEGF as multiplex mediators in articular cartilage degeneration.

## Introduction

Osteoarthritis (OA) is characterized by a breakdown of the extracellular matrix (ECM) of articular cartilage in the affected joints. The pathogenesis of OA involves multiple aetiologies, including mechanical, genetic and biochemical factors. However, the precise signalling pathways in the degradation of articular cartilage ECM and development of OA are still not fully understood. Several studies have demonstrated the involvement of cytokines, such as IL-1 and IL-6, or tumour necrosis factor (TNF)-α, in addition to proteases, such as matrix metalloproteases (MMPs), in the initiation and progression of articular cartilage destruction [1,2]. The imbalance between activated proteinases and inhibitors ultimately leads to an altered net proteolysis of cartilage components. Once damaged, articular cartilage has a poor capacity for intrinsic repair.

Angiogenesis, the development of new blood vessels by sprouting from pre-existing endothelium, is a significant component of a wide variety of biological processes [3,4]. However, in rheumatoid arthritis, new capillary blood vessels invade the joints from the emerging synovial pannus and aid in the destruction of articular cartilage [5], even in the absence of a causative factor. The most important mediator of angiogenesis is vascular endothelial growth factor (VEGF) [6], which stimulates capillary formation *in vivo *and has direct mitogenic actions on various cells *in vitro *[7]. Recent data reveal expression of VEGF in OA cartilage and reflect the ability of VEGF to enhance catabolic pathways in chondrocytes by stimulating MMP activity and reducing natural MMP inhibitors, that is tissue inhibitors of MMPs (TIMPs) [8-11]. These data suggest that, except from the effect of VEGF on proliferation of synovial membranes, chondrocyte-derived VEGF promotes catabolic pathways in the cartilage itself, thereby leading to a progressive breakdown of the ECM of articular cartilage.

Recent investigations have revealed the participation of free radicals in the pathogenesis of articular cartilage degradation [12]. Free radicals are highly reactive in oxidative processes and are essentially involved in physiological reactions, such as the cellular respiratory chain. However, uncontrolled release of free radicals can result ultimately in an imbalance, with respect to their inhibitors or antioxidants. Moreover, free radicals can stimulate inflammatory pathways or damage lipids, proteins or DNA [13]. In the nomenclature of free radicals, the term 'reactive oxygen species' (ROS) has prevailed, although ROS can be differentiated into reactive nitrogen species and other oxidant species. The relationship between ROS and articular cartilage degradation is complex and involves multiple pathways [14]. ROS can induce changes in biosynthetic activity [15], in addition to apoptosis [16]. In addition, ROS can influence transcription factors in chondrocytes and induce the expression of catabolic cytokines [17]. However, the evidence for the role of ROS is conflicting, because other investigators have demonstrated anti-inflammatory properties of ROS in articular cartilage [18].

The relationship between ROS and VEGF in articular cartilage degradation has not been investigated previously. Investigations focusing on the effects of ROS have used ROS donors as stimulants. Phorbol myristate acetate (PMA) activates protein kinase C and upregulates nicotinamide adenine dinucleotide phosphate (NADPH) oxidase, leading to enhanced production of superoxide anions (O_2_^.-^) [19], one of the major ROS. Another potent ROS donor is 3-morpholino-sydnonimine hydrochloride (SIN-1), which spontaneously decomposes to nitric oxide (NO) radicals and O_2_^.- ^[20].

Therefore, the present study was performed to investigate the expression of VEGF, VEGF-121 and VEGF-165 splice variants and VEGF receptors (VEGFR-1 and VEGFR-2) after stimulation by ROS donors. Knowledge of this expression could lead to a better understanding of the complex role of ROS and VEGF in articular cartilage degeneration.

## Materials and methods

### Reagents

Chemical reagents were purchased from Sigma (Munich, Germany), unless otherwise indicated.

### Synovial fluid

Samples of synovial fluid from patients with OA (*n *= 8) were obtained from the Department of Orthopaedic Surgery at the University Hospital Schleswig-Holstein (Kiel, Germany). Synovial fluid from healthy joints was collected from deceased donors (*n *= 5) at the Department of Anatomy, Christian-Albrechts-University (Kiel, Germany).

### Chondrocyte monolayer culture

The human C28/I2 chondrocyte cell line was used in monolayer culture. These chondrocytes, which were immortalized using SV-40 large T-antigen, continue to express chondrocyte-specific aggrecan and collagen type II mRNA after multiple subculture [21]. Chondrocytes were seeded (500,000 cells/25 cm^2^) in DMEM supplemented with 10% foetal bovine serum, 10 mM h-(2-hydroxyethyl)-1-piperazinethansulfonacid (HEPES) buffer, 1 mM sodium pyruvate, 0.4 mM proline, 20 μg/ml ascorbic acid, 100 U/ml penicillin G, 100 μg/ml streptomycin and 0.25 μg/ml amphotericin B. After reaching 80% confluence, the cells were rinsed twice with HANK's solution and placed in serum-free medium, containing 0.05% BSA, for subsequent stimulation.

### Human articular cartilage explants

The tissue harvest was approved by the Ethical Commission of Christian-Albrechts-University of Kiel (Kiel, Germany). Knee cartilage was obtained from the Institute of Pathology, Christian-Albrechts-University, (Kiel, Germany) as post-mortem donor tissue. Donors who were 75 years of age or older were excluded; the average age of the donors was 54 years. Knee cartilage was scored, using a modified Collins scale [22], for visual degeneration of grade 2 or less, otherwise the sample was rejected.

Cartilage–bone cylinders (11 mm in diameter) from the femoral condyles and femoropatellar groove were punched out perpendicular to the cartilage surface using Arthrex^® ^(Arthrex GmbH, Karlsfeld, Germany) instruments for osteochondral transplantation (T-handle bar and punch; AR-1980D-11). The cartilage–bone samples were removed, rinsed in HANK's solution (supplemented with antibiotics; see below) and placed in a microtome holder. After creating a level surface by removing superficial tissue, the cartilage tissue was sliced at a thickness of 1 mm. Finally, up to eight explant discs (measuring 3 mm in diameter and 1 mm in thickness) were punched from each slice. In all subsequent experiments, treatment groups were location-matched by distributing the explant discs from a single slice to each of the different groups.

Cartilage explants were equilibrated for 2 days in culture medium (250 μl of high-glucose DMEM with supplements (as above) per explant in a 96-well plate) under free-swelling conditions at 37°C in a standard cell-culture environment. Then, cartilage explants were rinsed twice with HANK's solution and placed in serum-free medium, containing 0.05% BSA, for subsequent stimulation. For each group, we used eight cartilage explants in five different experiments.

### Stimulants

PMA was used at concentrations of 5, 10 and 20 μg/ml in medium. SIN-1 concentrations were 1, 10 and 20 μM. Chondrocytes in monolayer culture were exposed to PMA or SIN-1 stimulation for 48 hours and cartilage explants were similarly stimulated for 72 hours.

### Isolation of RNA and cDNA synthesis

Total RNA was extracted from immortalized chondrocytes using an RNeasy Total RNA Kit (Qiagen, Hilden, Germany). Total RNA from tissue homogenates of cartilage explants was extracted using the TriZOL Reagent (Invitrogen, Life Technologies, Karlsruhe, Germany). DNA contamination was destroyed by digestion with RNase-free DNase-I (20 minutes at 25°C; Boehringer, Mannheim, Germany), and cDNA was generated from 100 ng RNA reacting with 1 μl (20 pmol) of oligo(dT)_15 _primer (Amersham Biosciences, Amersham, UK) and 0.8 μl of superscript RNase H-reverse transcriptase (Gibco, Paisley, UK) in 50 μl total volume for 60 minutes at 37°C. For each sample, a control without reverse transcriptase was run in parallel to enable assessment of genomic DNA contamination.

### RT-PCR for VEGF splice variants

For PCR, 4 μl of cDNA was incubated with 30.5 μl water, 4 μl 25 mM MgCl_2_, 1 μl deoxynucleoside-triphosphate, 5 μl 10 × PCR buffer, 0.5 μl (2.5 U) Platinum *Taq *DNA polymerase (Gibco) and 2.5 μl (10 pmol) of each primer pair. The following primers and conditions were applied: VEGF splice variants, 5'-CCA-TGA-ACT-TTC-TGC-TGT-CTT-3' (sense) and 5'-TCG-ATC-GTT-CTG-TAT-CAG-TCT-3' (antisense), with 40 cycles performed at a 55°C annealing temperature. A glyceraldehyde-3-phosphate-dehydrogenase (G3PDH)-specific primer pair (5'-ATC-AAG-AAG-GTG-GTG-AAG-CAGG-3' (sense) and 5'-TGA-GTG-TCG-CTG-TTG-AAG-TCG-3' (antisense), with 40 cycles at 58°C) served as the internal control (983 bp).

### Quantitative real-time RT-PCR for VEGF, VEGFR-1 and VEGFR-2

Real-time RT-PCR was carried out using a one-step system, according to the manufacturer's instructions (QuantiTect SYBR Green RT-PCR; Qiagen), with 100 ng of total RNA in an i-Cycler (Biorad, Munich, Germany). The temperature profile included an initial denaturation for 15 minutes at 95°C, followed by 37 cycles of denaturation at 95°C for 15 seconds, annealing at a temperature of 60°C for 30 seconds, elongation at 72°C (the elongation time depended on the size of the fragment, that is the number of bp divided by 25 yielded the time in seconds) and fluorescence monitoring at 72°C. Each cDNA sample was analysed for expression of the gene of interest, in addition to G3PDH, with the fluorescent TaqMan 5'-nuclease assay, using 2 × TaqMan Master Mix (Applied Biosystems, Foster City, CA, USA) and 20 × assay-on-demand TaqMan primers and probes in a total volume of 20 μl. Each plate included no-template controls (NTCs). TaqMan human primers and probes had the following identification numbers: VEGF, Hs00173626_m1; VEGFR-1, Hs00176573_m1; VEGFR-2, Hs00176676_m1; and G3PDH, Hs99999905_m1. The cycle of threshold (C_T_) for each sample was averaged and normalized to G3PDH. The results were then analysed by comparative ΔΔC_T _method (2^(-ΔΔCT)^) for relative quantification of gene expression:

ΔΔC_T _= ΔC_T _(sample) - ΔC_T _(control)

ΔC_T _(sample) = C_T _(sample; target) - C_T _(sample; G3PDH)

ΔC_T _(control) = C_T _(control; target) - C_T _(control; G3PDH)

### ELISA

After stimulation with PMA or SIN-1, the conditioned medium supernatant of each chondrocyte monolayer or cartilage explant culture was collected. Aliquots were analysed using a sandwich ELISA (R&D Systems, Minneapolis, MN, USA) to detect VEGF, and signals were identified by a chemoluminescence reaction (ECL-Plus; Amersham-Pharmacia, Uppsala, Sweden). Human recombinant VEGF^165 ^(Repro Tech, Rocky Hill, NJ, USA) served as an internal standard. Aliquots of synovial fluid samples from OA patients were analysed by an identical procedure. VEGF concentrations were normalized using Bradford reagent (Roti-Quant; Roth, Karlsruhe, Germany).

### Biochemical analysis

Concentrations of nitrite, the stable end product of NO, were analysed in the culture medium using Griess reagent, according to the protocol described by Ailland and coworkers [23]. Results were corrected for the nitrite content of pure medium with or without (blanks) the PMA or SIN-1 stimulants. Data were calculated according to the amount of medium and normalized to the number of cells or cartilage wet weight (monolayer or tissue explants, respectively) and control group, which was set at 100%.

### Statistics

All data are shown as mean ± standard error of the mean (SEM), unless indicated otherwise. Differences between analysed data were tested using the Student *t *test. Significance was set to p value of < 0.05.

## Results

### Increased levels of VEGF in synovial fluids from patients with OA

**Figure 1:**
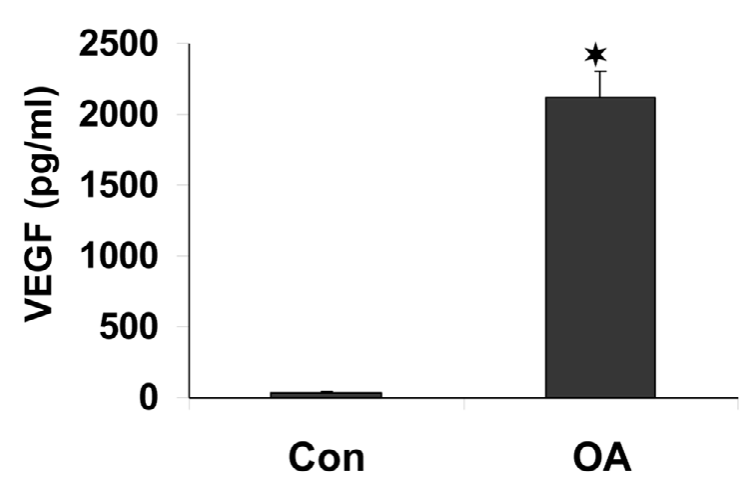
Comparison of the VEGF content of synovial fluid (as determined by ELISA) from healthy (Con) or OA patients. The level of VEGF is strongly increased in the synovial fluid of OA patients. Results are shown as mean ± standard error of the mean; *n *= 5 (Con) and *n *= 8 (OA). * p < 0.05. Con, control; OA, osteoarthritis; VEGF, vascular endothelial growth factor.

To characterize VEGF *in vivo*, aspirates of synovial fluid were assessed for VEGF by ELISA. Compared with VEGF concentrations in healthy joints (36 pg/ml), VEGF concentrations in the synovial fluid of patients with OA were significantly higher (2,100 pg/ml, which was nearly 60-fold higher than healthy synovial fluids; control versus OA, p ≤ 0.05 (Figure 1)).

### The VEGF splice variants VEGF-121 and VEGF-165 are detectable by splice-variant RT-PCR

**Figure 2:**
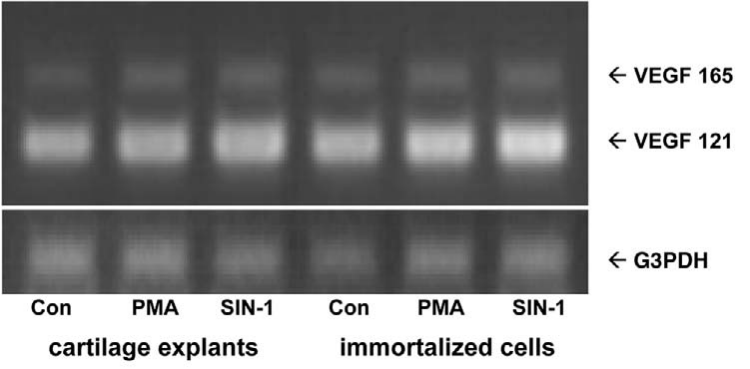
VEGF splice variants. Expression of VEGF-121 (526 bp) and VEGF-165 (658 bp) in cartilage explants and immortalized chondrocytes after stimulation with PMA (10 μg/ml) and SIN-1 (10 μM). The splice variants VEGF-121 and VEGF-165 are detected in cartilage explants and C28/I2 cells. Con, control; PMA, phorbol myristate acetate; SIN-1, 3-morpholino-sydnonimine hydrochloride; VEGF, vascular endothelial growth factor.

To determine whether the splice variants VEGF-121 (526 bp) and VEGF-165 (658 bp) are expressed after stimulation of chondrocytes with PMA, a known inducer of O_2_^.-^, semiquantitative RT-PCR was performed (Figure 2). Both splice variants, VEGF-121 (526 bp) and VEGF-165 (658 bp), are present in immortalized chondrocytes and articular cartilage explants. There were bold signals corresponding to VEGF-121 and fine bands corresponding to VEGF-165. In general, more intense signals were present in the chondrocytes compared with those in cartilage explants. Stimulation with PMA (10 μg/ml) increased the signals of the mRNAs encoding the VEGF splice variants in monolayer chondrocytes and cartilage explants.

### Real-time RT-PCR revealed upregulation of VEGF, VEGFR-1 and VEGFR-2 mRNA after stimulation with ROS donors

**Figure 3:**
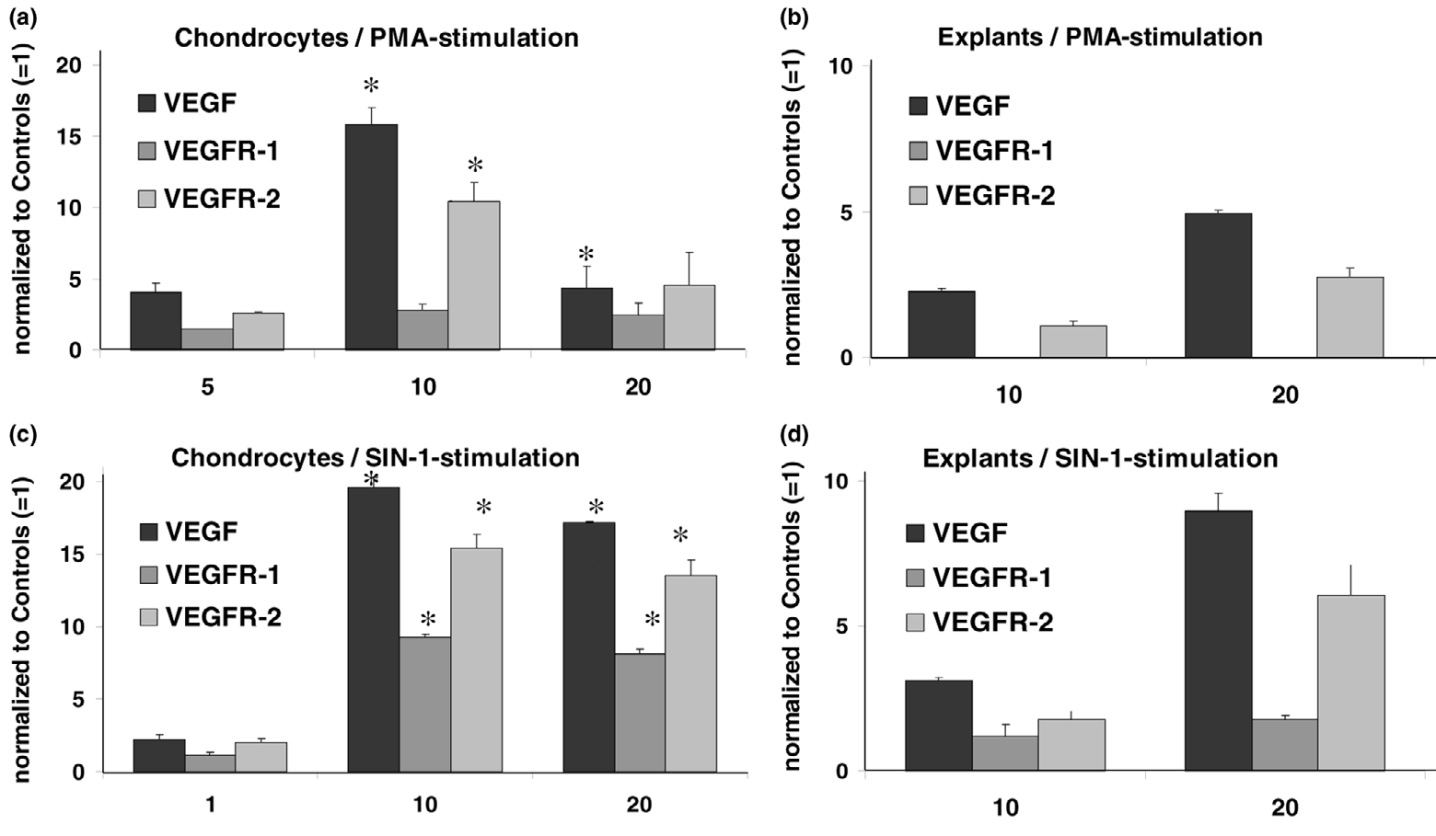
Quantitative mRNA expression of VEGF, VEGFR-1 and VEGFR-2 normalized to the control (*n *= 1). Stimulation of immortalized chondrocytes **(a) **and **(c) **and articular cartilage explants **(b) **and **(d) **with PMA (μg per 1 ml of medium) or SIN-1 (μM). VEGFR-1 is undetectable in **(b)**. mRNA expression of VEGF, VEGFR-1 and VEGFR-2 is upregulated after stimulation with reactive oxygen species donors. Results are shown as mean ± standard error of the mean for five separate experiments. * p < 0.05 versus control. G3PDH, glyceroaldehyde-3-phosphate dehydrogenase; PMA, phorbol myristate acetate; SIN-1, 3-morpholino-sydnonimine hydrochloride; VEGF, vascular endothelial growth factor; VEGFR, VEGF receptor.

The levels of mRNA encoding VEGF and VEGF receptors (VEGFR-1 (flt-1) and VEGFR-2 (KDR, flk-1)) were quantified by real-time RT-PCR. After challenging with PMA, VEGF mRNA was upregulated in monolayer chondrocytes (Figure 3a). The levels of VEGF were elevated dose-dependently, from 4.1-fold at 5 μg of PMA to 15.8-fold at 10 μg of PMA, with a following decrease by 4.4-fold at 20 μg of PMA. Although VEGFR-1 mRNA levels were increased only slightly (1.5-fold, 2.8-fold and 2.4-fold) after PMA stimulation (at 5, 10 and 20 μg, respectively), the levels of VEGFR-2 mRNA were elevated 2.6-fold, 10.4-fold and 4.6-fold at PMA concentrations of 5, 10 and 20 μg, respectively. Stimulation of cartilage explants with 10 and 20 μg of PMA upregulated VEGF mRNA by 2.3-fold and 4.9-fold, respectively, compared with the control (Figure 3b). The level of VEGFR-2 mRNA was unaffected by 10 μg of PMA and increased 2.7-fold by 20 μg of PMA. The level of VEGFR-1 mRNA was undetectable in cartilage explants after treatment with PMA.

Treatment of chondrocytes (monolayer) with SIN-1 resulted in responses similar, in part, to those after PMA stimulation. After treatment with 1, 10 and 20 μM of SIN-1, VEGF mRNA expression was increased 2.2-fold, 19.6-fold and 17.2-fold, respectively (Figure 3c), and VEGFR-2 mRNA expression was elevated 2.0-fold, 15.4-fold and 13.5-fold, respectively. In contrast to PMA, 1, 10 and 20 μM of SIN-1 enhanced VEGFR-1 mRNA levels 1.2-fold, 9.2-fold and 8.1-fold, respectively, compared with the control. In the cartilage explants, 3.1-fold and 9.0-fold increases in VEGF mRNA expression were apparent after treatment with 10 and 20 μM of SIN-1, respectively (Figure 3d). VEGFR-2 mRNA expression was increased by 1.8-fold and 6.1-fold at 10 and 20 μM of SIN-1, respectively. In contrast to the undetectable level of VEGFR-1 in cartilage explants after PMA treatment, VEGFR-1 mRNA levels slightly increased after treatment with SIN-1 (1.2-fold and 1.8-fold increases at 10 and 20 μM of SIN-1, respectively).

### Enhanced VEGF production after stimulation with PMA or SIN-1 (using ELISA)

**Figure 4:**
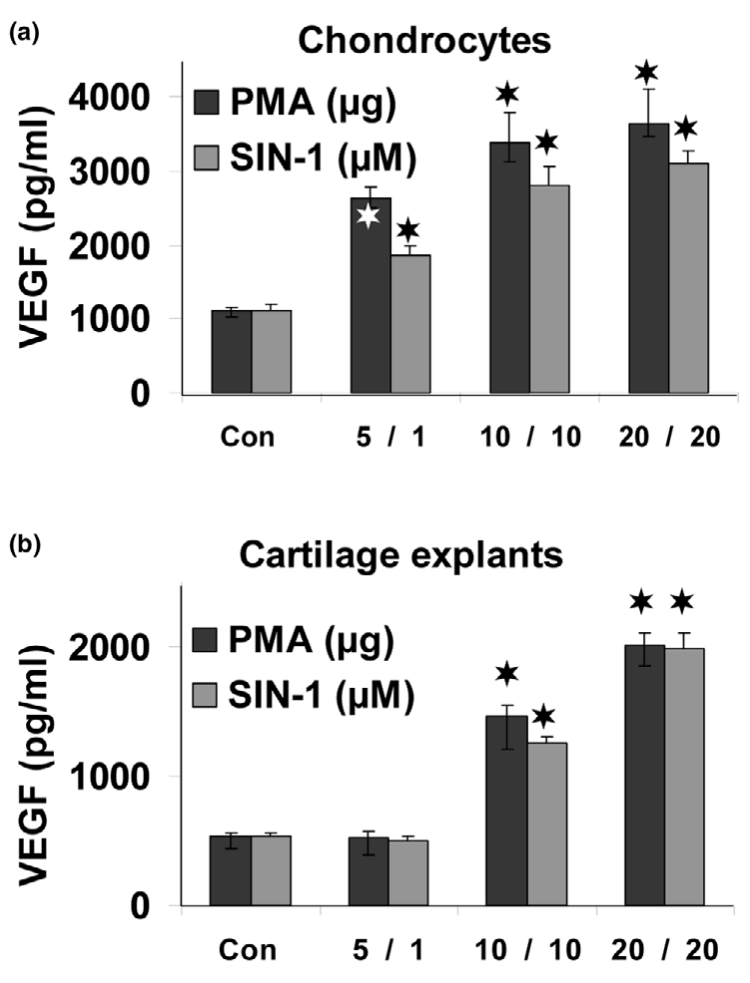
The VEGF content of medium supernatant (as determined by ELISA). Stimulation of immortalized chondrocytes **(a) **and articular cartilage explants **(b) **with PMA (μg per 1 ml of medium) or SIN-1 (μM). The level of VEGF protein is increased after stimulation with PMA or SIN-1. Results are shown as mean ± standard error of the mean for five separate experiments. * p < 0.05 versus Con. Con, control; PMA, phorbol myristate acetate; SIN-1, 3-morpholino-sydnonimine hydrochloride; VEGF, vascular endothelial growth factor.

To determine whether the increased levels of VEGF mRNA were reflected in the production of protein by chondrocytes, an ELISA was performed to quantify the VEGF content in the medium supernatants (Figure 4). Chondrocyte monolayer controls released 1,100 pg of VEGF per 1 ml of medium and cartilage explant controls released 540 pg of VEGF per 1 ml of medium. Treatment with PMA at concentrations of 5, 10 and 20 μg/ml increased VEGF production by 2.4-fold, 3.0-fold and 3.4-fold, respectively, in monolayer chondrocytes (control versus PMA, p ≤ 0.05) and treatment with SIN-1 at concentrations of 1, 10 and 20 μM increased the level of VEGF by 1.7-fold, 2.5-fold and 2.8-fold, respectively (control versus SIN-1, p ≤ 0.05; Figure 4a). In cartilage explants, no increase in VEGF was detected at 5 μg of PMA or 1 μM of SIN-1 (Figure 4b). Using higher concentrations, VEGF production was increased 2.7-fold and 3.8-fold after treatment with PMA at concentrations of 10 and 20 μg, respectively (control versus PMA, p ≤ 0.05), and 2.4-fold and 3.7-fold after treatment with SIN-1 at concentrations of 10 and 20 μM, respectively (control versus SIN-1, p ≤ 0.05).

### Increasing nitric oxide content of the medium supernatant after stimulation with ROS donors

**Figure 5:**
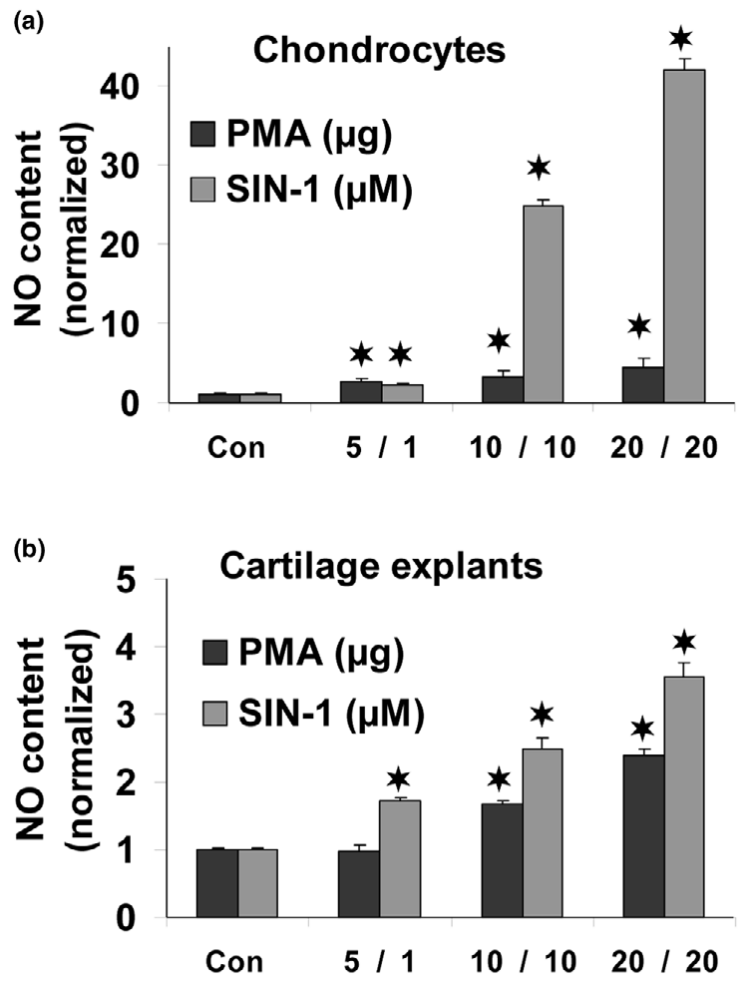
The NO content of medium supernatant normalized to the Con (*n *= 1). Stimulation of immortalized chondrocytes **(a) **and articular cartilage explants **(b) **with PMA (μg per 1 ml of medium) or SIN-1 (μM). The level of NO is increased after stimulation with reactive oxygen species donors. Results are shown as mean ± standard error of the mean for five separate experiments. * p < 0.05 versus Con. Con, control; NO, nitric oxide; PMA, phorbol myristate acetate; SIN-1, 3-morpholino-sydnonimine hydrochloride.

PMA dose-dependently increased the NO content of culture medium from chondrocytes and, to a lesser extent, cartilage explants. In monolayer cultures, NO levels were increased 2.6-fold, 3.2-fold and 4.4-fold at 5, 10 and 20 μg/ml of PMA compared with the control (control versus PMA, p ≤ 0.05; Figure 5a). By contrast, the cartilage explants showed no response to low-dose PMA stimulation (5 μg) and only 1.7-fold and 2.4-fold increases using 10 and 20 μg of PMA, respectively (control versus PMA, p ≤ 0.05; Figure 5b). After treatment with SIN-1, the NO content showed similar results but the effect was more extended (Figure 5a): in monolayer chondrocytes, the NO content was 2.1-fold, 24.8-fold and 41.9-fold higher than control after treatment with 1, 10 and 20 μM of SIN-1 (control versus SIN-1, p ≤ 0.05). In the cartilage explants, the effects were attenuated compared with the monolayer cultures, with 1.7-fold, 2.5-fold and 3.6-fold increases in NO content reported after treatment with 1, 10 and 20 μM of SIN-1, respectively (control versus SIN-1, p ≤ 0.05; Figure 5b).

## Discussion

Our findings show that the level of VEGF in synovial joint fluids from patients suffering from OA is 60-fold higher than healthy joints. Moreover, our *in vitro *model revealed that VEGF mRNA and protein levels and VEGF receptors are increased by PMA or SIN-1 stimulation in chondrocytes and human articular cartilage explants. We conclude that the presence of ROS, or activation of production of O_2_^.-^, is responsible for the observed results. Thus, VEGF accumulation in the synovial fluid is, at least in part, cartilage-derived.

Inflammation in OA is known to be associated with activation of host angiogenesis [24]. VEGF is one of the most potent proangiogenic stimuli of neovascularization. Furthermore, the capacity of VEGF to mediate chemotaxis, raise vascular permeability for neutrophil influx and activate MMPs in chondrocytes suggests a central function in catabolic pathways of OA joints [9,25]. In addition to the involvement of VEGF in the development of OA, VEGF has biological importance in cartilage metabolism during rheumatoid arthritis. The initial growth and invasion of the synovial pannus tissue contributes to the subsequent cartilage destruction. Blockade of VEGFR-I resulted in reduced intensity of clinical manifestations and prevented joint destruction in a mouse model of rheumatoid arthritis [26]. It is obvious that tissues other than cartilage are participating in these processes, especially the surrounding synovial tissue. This reflects the findings of Felson and coworkers [27], who declared OA to be a disease involving the whole joint. In summary, these data support the role of VEGF in mediating destructive processes in articular joints and encouraged us to investigate the relationship between VEGF expression and ROS in articular cartilage. Focusing on other cell types, such as glomerular podocytes, endothelial cells and skeletal muscle fibres, a correlation between ROS and VEGF is described, but the results were contradictory [28,29].

Here, we demonstrate increased VEGF mRNA expression and production in cultured human chondrocytes and articular cartilage explants after challenge by ROS donors. We conclude that the observed increase in VEGF content in synovial fluid of OA joints is produced partly by articular chondrocytes and consistent with previous findings from this and other laboratories, which showed an increase in VEGF content in OA cartilage [8,10,11]. The observation that the concentration of VEGF is positively correlated to joint destruction and vascularization of synovial membrane in rheumatoid arthritis [30] suggests the potential impact of VEGF in the pathophysiology of OA.

Our demonstration of ROS-mediated induction of the angiogenic factor VEGF in human chondrocytes and articular cartilage explants is consistent with prior reports showing that NO stimulates VEGF production in chondrocytes [31]. However, we observed different responses to the two ROS donors. The NO content after stimulation with SIN-1 was up to tenfold higher than after stimulation with PMA and only SIN-1 induced upregulation of VEGFR-1 mRNA. It could be speculated that PMA induces enhanced production of O_2_^.- ^within the cell, whereas SIN-1 spontaneously decomposes to O_2_^.-^. A possible mechanism by which PMA increases the level of VEGF is the activation of AP-1 (activator protein 1), with subsequent activation of the TPA (12-O-tetradecanoylphorbol-13-acetate) element of the VEGF promoter. The mode of action of SIN-1 is presumably by an increase in HIF-1α (hypoxia inducible factor subunit alpha). Future studies are necessary to investigate the signal transduction pathways of PMA and SIN-1 in chondrocytes.

## Conclusion

By amplifying distinct ROS-dependent destructive pathways in cartilage and joints, VEGF seems to have a crucial role in the degeneration of articular cartilage by promoting neoangiogenesis in the emerging synovial tissue and stimulating cartilage matrix-degrading pathways. Interestingly, do the splice variants of young donors differ from those of old donors? VEGF-121 and VEGF-165 have high angiogenic potencies and lead to a rapid and strong invasion of blood vessels. Therefore, in the long term, a new understanding of the mechanisms responsible for cartilage destruction in OA might enable the development of novel strategies for intervention and treatment. The functional knockout of VEGF with, for example, soluble receptors might be potential therapeutic target for the treatment of OA.

## Abbreviations

AP-1 = activator protein 1; DMEM = Dulbecco's modified Eagle's medium; ECM = extracellular matrix; ELISA = enzyme-linked immunosorbent assay; G3PDH = glyceroaldehyde-3-phosphate dehydrogenase; IL = interleukin; MMP = matrix metalloproteinases; NO = nitric oxide; NTC = no-template control; OA = osteoarthritis; PMA = phorbol myristate acetate; ROS = reactive oxygen species; RT-PCR = reverse transcriptase polymerase chain reaction; SEM = standard error of the mean; SIN-1 = 3-morpholino-sydnonimine hydrochloride; TIMP = tissue inhibitor of matrix metalloproteinase; TNF = tumour necrosis factor; TPA = 12-O-tetradecanoylphorbol-13-acetate; VEGF: vascular endothelial growth factor; VEGFR = VEGF receptor.

## Competing interests

The authors declare that they have no competing interests.

## Authors' contributions

JV, DV, CW, BK and TP performed the experiments and contributed to the draft manuscript; DV and TP contributed equally to the present work. MG established the C28/I2 cell line, designed part of the study and contributed to the draft manuscript. The manuscript has been read and approved by all authors.
